# An Unexpected Complication after Headless Compression Screw Fixation of an Osteochondral Fracture of Patella

**DOI:** 10.1155/2016/7290104

**Published:** 2016-03-14

**Authors:** Suavi Aydoğmuş, Tahir Mutlu Duymuş, Tolga Keçeci

**Affiliations:** Department of Orthopedic Surgery and Traumatology, Haydarpaşa Numune Education and Research Hospital, Uskudar, 34668 Istanbul, Turkey

## Abstract

This study evaluated complications associated with implant depth in headless compression screw treatment of an osteochondral fracture associated with a traumatic patellar dislocation in a 21-year-old woman. Computed tomography and X-rays showed one lateral fracture fragment measuring 25 × 16 mm. Osteosynthesis was performed with two headless compression screws. Five months later, the screws were removed because of patella-femoral implant friction. We recommend that the screw heads be embedded to a depth of at least 3 mm below the cartilage surface. Further clinical studies need to examine the variation in cartilage thickness in the fracture fragment.

## 1. Introduction

Osteochondral fractures of the patella may follow traumatic dislocations and can often be missed on the first radiographs taken after trauma [1]. Osteochondral injuries are caused by the shear stress that develops with lateral dislocation of the patella striking the medial femoral condyle and subsequent relocation [2]. The treatment ranges from fracture fragment resection to fixation using different methods [3, 4]. However, there is a noticeable lack of information on the depth that headless compression screws should be embedded below the cartilage surface and the associated postoperative complications.

This case report discusses the use of headless compression screws in the surgical treatment of patellar osteochondral fractures together with points requiring attention in the surgical technique.

## 2. Case Presentation

**Figure 1:**
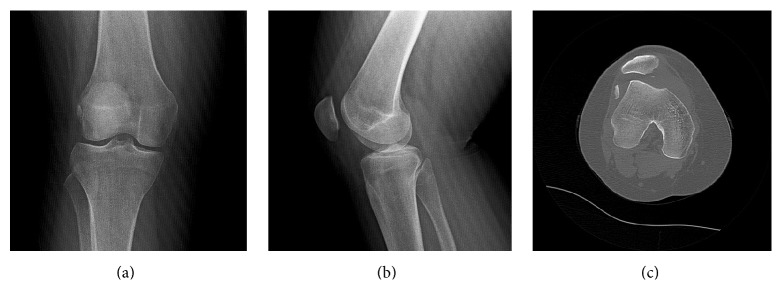
Preoperative anteroposterior (a) and lateral (b) X-rays and axial computed tomography (CT) (c) of the right knee show the free fragment and osteochondral defect.

A 21-year-old woman presented to the Emergency Service with pain and swelling of the right knee after twisting the knee while playing sport. The physical examination showed an effusion in the knee, sensitivity on palpation, and an inability to reach full extension. A direct radiograph showed a free bone fragment lateral to the lateral femoral condyle (Figures 1(a) and 1(b)). Computed tomography (CT) showed an osteochondral defect in the patella inferomedially and a free fragment at the level of the lateral femoral condyle (Figure 1(c)). Obviously, the flake fracture was due to patella dislocation. The free fragment measured 25 × 16 mm; the bone was 4 mm thick and the cartilage was 3 mm thick. An intra-articular hematoma was aspirated and a long leg splint was applied with ice compression and elevation until surgery.

**Figure 2:**
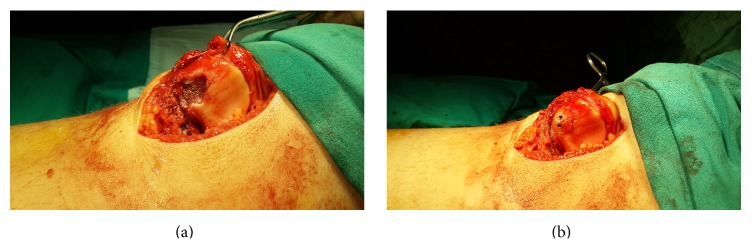
Osteochondral defect area after debridement (a), osteosynthesis with two headless compression screws (b).

Spinal anesthesia was administered and, with the patient in a supine position, a pneumatic tourniquet was applied to the right leg. With a medial parapatellar incision, an arthrotomy was made and the osteochondral defect was explored. A 100-mL hematoma was drained and the joint was washed with saline. The patellar chondral defect and the ends of the fracture fragment were debrided (Figure 2(a)). The fracture was then reduced and osteosynthesis was applied with two headless compression screws (Figure 2(b)). Medial retinaculum was repaired primarily because it was incised during medial arthrotomy. The tight lateral side was loosened and when the patella appeared stable, the surgical procedure was terminated. After inserting a drain and applying a dressing, the operation was ended.

The patient was put in a long leg splint postoperatively. On the second day, the splint was exchanged with immobilizing brace, full weight-bearing was permitted, and quadriceps exercises were started. After 2 weeks, the degree of flexion was increased gradually and, in the 6th week, the brace was removed and open-chain exercises were continued. Four months postoperatively, full healing was seen clinically and radiologically and there was full-joint range of movement.

**Figure 3:**
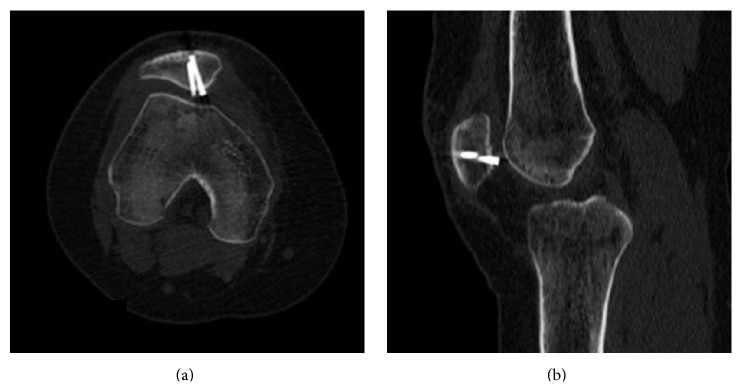
CT showing the relationship between the patellofemoral joint and implant: axial (a) and sagittal (b) slices.

**Figure 4:**
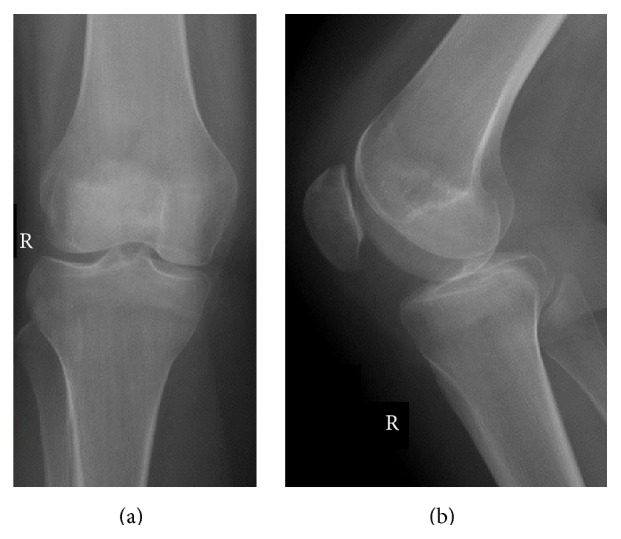
Anteroposterior (a) and lateral (b) follow-up X-rays after the second operation.

Five months postoperatively, the patient complained of stiffness in the knee and mild pain. The physical examination detected a feeling of friction under the anterior patella at early degrees of flexion. The patient's Knee injury and Osteoarthritis Outcome Score (KOOS) was 71; the score was limited mainly because of loss of function during high level activities. CT showed full healing at the osteotomy line (Figure 3). It was thought that friction caused by the headless compression screws in the patellofemoral joint had led to cartilage destruction, so we decided to remove the screws. Intraoperatively, the fragment was stable and the cartilage surface was intact, but screws were palpable below the cartilage, so both screws were removed. There were no signs of screw loosening. Postoperatively, quadriceps strengthening exercises were started. Two months later, the final follow-up radiographs were evaluated as normal (Figure 4), the joint range of movement was full, all of the complaints had resolved, and the KOOS was 91.

## 3. Discussion

While patellar osteochondral fractures were once thought rare, advanced diagnostic techniques show that they are actually more frequent. In 1976, Rorabeck and Bobechko reported osteochondral fractures in 5% of patella dislocations, while in 2003 Nomura et al. reported cartilage injuries in 95% of acute patellar dislocations and cartilage defects (osteochondral or chondral fracture) in 72% based on diagnostic arthroscopy [5, 6].

Different fixation methods have been described in the surgical treatment of patellar osteochondral fractures. One study obtained good results using a two-component fibrin sealant (fibrin adhesive system) in two patients with large osteochondral fragments, while another study reported good results with butyl-2-cyanoacrylate tissue glue [7, 8]. Fixation methods with bone pegs and autologous bone grafts taken from the proximal tibia or midshaft are treatment alternatives and these increased blood circulation in the region of the bone grafts and strengthened healing [9, 10]. Different surgical options include the use of bioabsorbable ultra-high-strength poly(L-lactide) pins or absorbable sutures (Vicryl 2) with good long-term functional results [11–13].

Similar to our case, Rae and Khasawneh successfully used headless cannulated compression screws for large fragments [14]. As the screws are headless, local friction and irritation are prevented by completely embedding these screws in the cartilage and bone. Consequently, we selected headless cannulated compression screws to fix the fracture fragment in our patient as it was thought that greater stability would be obtained given the large size of the fragment. While the desired positive results were achieved in terms of fixation and osteosynthesis, friction in the early follow-up period led to pain and restriction in the patella-femoral joint, necessitating removal of the headless screws. Unlike other screws and pins [15–17], there is normally no need to remove headless cannulated screws, and there is no requirement for a second surgical intervention [14, 18]. In our patient, however, this advantage was not realized and a second operation was necessary.

It was thought that embedding the headless compression screws in the cartilage to a depth where they could not be palpated prevented the possibility of friction. Technically, without measuring the distance that the screw was embedded, the evaluation was made subjectively by palpation. A literature review found no clear information on how deep headless cannulated screws should be embedded. For a scaphoid fracture, embedding the screw 2 mm was recommended [19] and, in a separate biomechanical study, the peak compression strength was observed at −3.1 mm [20]. However, the latter study did not consider the safety of embedding but only the peak compression strength. Following fixation of a traumatic chondral fracture of the femoral groove, Hatayama et al. observed that the chondrocyte arrangement was normal in the chondral fragment in the healing tissue, but the fragment volume was reduced [21]. Also it is possible that swelling of cartilage may occur due to trauma. In our case, there was no evidence of loosening or reduction loss in the early or late radiological evaluations. We concluded that a possible reduction in the cartilage fragment volume led to insufficient embedding of the screw head resulting in the friction. Therefore, we recommend embedding the screw at a depth of 3 mm. However, the surgeon may choose to embed it even deeper. Yet, he/she has to consider that the deeper the screw is embedded, the more the compression force decreases. But, in a separate biomechanical study, the peak compression strength was founded at −3.1 mm [20].

In conclusion, a headless cannulated compression screw is an appropriate choice for fixation and osteosynthesis in intra-articular fractures. However, the screws need to be embedded deeply below the cartilage surface or absorbable or adhesive fixation methods should be considered as alternatives. Further studies should examine postoperative changes in cartilage thickness and the necessary amount of embedding.
